# Prediction of thermal boundary resistance by the machine learning method

**DOI:** 10.1038/s41598-017-07150-7

**Published:** 2017-08-02

**Authors:** Tianzhuo Zhan, Lei Fang, Yibin Xu

**Affiliations:** National Institute for Materials Science, 1-2-1 Sengen, Tsukuba, 305-0047 Japan

## Abstract

Thermal boundary resistance (TBR) is a key property for the thermal management of high power micro- and opto-electronic devices and for the development of high efficiency thermal barrier coatings and thermoelectric materials. Prediction of TBR is important for guiding the discovery of interfaces with very low or very high TBR. In this study, we report the prediction of TBR by the machine learning method. We trained machine learning models using the collected experimental TBR data as training data and materials properties that might affect TBR as descriptors. We found that the machine learning models have much better predictive accuracy than the commonly used acoustic mismatch model and diffuse mismatch model. Among the trained models, the Gaussian process regression and the support vector regression models have better predictive accuracy. Also, by comparing the prediction results using different descriptor sets, we found that the film thickness is an important descriptor in the prediction of TBR. These results indicate that machine learning is an accurate and cost-effective method for the prediction of TBR.

## Introduction

Tailoring the thermal resistance of materials is vital for the thermal management of high power micro- and opto-electronic devices and for the development of high efficiency thermal barrier coatings and thermoelectric materials^1–3^. The overall thermal resistance of a material system is composed of the thermal resistance of the constituent materials and the thermal boundary resistance (TBR) between those materials. Heat in dielectric materials and semiconductors is transported predominantly by phonons, which undergo scattering in materials by interacting with defects, other phonons, boundaries, isotope, etc^4^. These processes cause the thermal resistance of the constituent materials. When heat passes through an interface between two materials, a temperature discontinuity occurs at the interface. TBR is defined as the ratio of the temperature discontinuity at an interface to the heat flux flowing across that interface. Typically, the characteristic length scales of the nanostructured materials are from several to hundreds of nanometers. By comparison, the phonon mean free paths (MFPs) in crystalline materials can be micrometers long^5^. When the characteristic length scales of nanostructured materials are shorter than the phonon MFPs, phonon transport is ballistic instead of diffusive. Furthermore, the number of interfaces increases greatly with decreasing characteristic length scales in nanostructured materials, such as superlattices. Thus, TBR can dominate the thermal resistance of the constituent materials and become a major component of the overall thermal resistance in nanostructured materials^1–3^. TBR is attributed to the mismatch between the phonon spectra of the materials on both sides of the interface^5^; interfacial properties, such as interfacial roughness, interdiffusion, bonding strength, and interface chemistry, also have a considerable effect^1–3^. Finding interfaces with very low or very high TBR experimentally is a high cost and time-consuming approach. Therefore, in order to guide the discovery of such interfaces, a reliable method for the prediction of TBR is necessary.

Several methods have been used for predicting TBR. The most common methods are the acoustic mismatch model (AMM) and the diffuse mismatch model (DMM)^6^. In the AMM, phonons is treated as plane waves, and the materials in which the phonons propagate are treated as continua. The transmission probabilities of phonons are calculated from the acoustic impedances on each side of the interface. A crucial assumption made in the AMM is that no scattering occurs at the interface. The assumptions of wave nature of phonon transport and specular scattering at the interface make the AMM valid when predicting TBR at low temperatures and at ideal interfaces. By comparison, in the DMM, completely diffuse scattering at the interface is assumed: a scattered phonon has no memory of its modes (longitudinal or transverse) and where it came from. The transmission probabilities of phonons are determined by the mismatch of the phonon density of states (DOS) on each side of the interface. Another crucial assumption made in the DMM is that phonons are elastically scattered: the transmitted phonons have the same frequencies with the incident phonons. These assumptions make the DMM invalid for an interface where inelastic phonon scattering occurs. In recent years, the molecular dynamics (MD) simulation method has emerged as a method for predicting TBR^7, 8^. In MD simulations, no assumptions concerning the nature of phonon scattering are required; the only required input to an MD simulation is the description of the atomic interactions. However, MD simulations also have limitations. The specification of the atomic interactions is typically done using empirical interatomic potential functions, and the results of MD simulations are realistic only when the specified atomic interactions mimic the forces experienced by the ‘real’ atoms^9^. Specifying an adequate description of atomic interactions that is applicable to various systems is a challenging task. Furthermore, MD simulations are computationally expensive and time-consuming, especially for simulation cells containing a large number of atoms. Thus, it is imperative to develop an accurate and cost-effective method to guide the discovery of interfaces with very low or very high TBR. In this work, we demonstrate a machine learning method for predicting TBR with much better predictive accuracy than the commonly used AMM and DMM.

## Methods

Machine learning is a subfield of computer science that allows computers to improve their performance through experience^10^. When using machine learning to make predictions, one should consider three key ingredients: training data, descriptors, and machine learning algorithms. In this work, the training data include a large amount of experimental TBR data collected from 62 published papers^1–3, 11–69^. The data consist of a total of 876 TBRs measured for 368 interfaces as a function of measurement temperature and other conditions. The 368 interfaces comprise 45 different materials. The descriptors are measurement temperature, film thickness, heat capacity, thermal conductivity, Debye temperature, melting point, density, speed of sound (longitudinal and transverse), elastic modulus, bulk modulus, thermal expansion coefficient, and unit cell volume; collectively, these form the set which we define as “all collected descriptors”. We chose these descriptors from two points of view. Firstly, the descriptors are the measurement conditions and the properties of the materials constituting the interfaces that might affect TBR. Secondly, the descriptors should be easily collected. Namely, the values of the descriptors are clearly given in the references. The data of the descriptors are collected from references including the Internet, published papers^1–3, 11–69^, and databases, such as Atom Work in the NIMS MatNavi database^70^ and the TPRC data series^71^. The descriptor data for all the materials and their origins are shown in the supplementary files. The machine learning algorithms used in this work and the model fitting process are briefly introduced in the following paragraphs.

### Generalized linear regression (GLR)

Linear regression is a method describing a linear relationship between a response and one or more descriptors^72^. The simple linear regression model can be described as

1.1 $$E({y}_{i})={\mu }_{i}={x}_{i}^{T}\beta ,\,i=1,\ldots ,N.$$

GLR extends simple linear regression. Unlike the simple linear regression, whose response has a normal distribution, the GLR response could be normal, binomial, Poisson, or something else. In GLR, a link function *f* is applied to the linear description, so the GLR model becomes

1.2 $$f({\mu }_{i})=f(E({y}_{i}))={x}_{i}^{T}\beta .$$

In our TBR prediction analysis, we choose the logarithm as the link function, $\mathrm{log}(E({y}_{i}))={x}_{i}^{T}\beta$, and the response has a Poisson distribution.

### Least-absolute shrinkage and selection operator regularization (LASSO-GLR)

LASSO regularization is a shrinkage method that imposes a penalty term to limit a model’s complexity^73^. It can identify important descriptors, select descriptors among redundant descriptors, and produce fewer coefficients in the model formula. Moreover, it can address descriptor multicollinearity. LASSO regularization in GLR is an extension of simple LASSO regularization. It is defined by

1.3 $$\mathop{min}\limits_{{\beta }_{0},\beta }(\frac{1}{N}Deviance({\beta }_{0},\beta )+\lambda \sum _{j=1}^{p}|{\beta }_{j}|).$$

where the second term with coefficient *λ* is a penalty term to balance model accuracy and complexity, and *Deviance* is the deviance between the response and the model fit by *β*_*0*_ and *β* coefficients. In this study, the response has a Poisson distribution, and the link function is logarithm. The model is first given with all collected descriptors. After training, the number of coefficients in model is reduced due to the LASSO screening.

### Gaussian process regression (GPR)

The GPR model is a nonparametric probabilistic model^74^. The goal of the GPR is to find a probabilistic distribution of new output given the training data and new input data, $P({y}_{new}|{y}_{train},{x}_{train},{x}_{new})$. Given that a latent variable *f*(*x*_*i*_) is a Gaussian process, then the joint distribution of a finite number of latent variables is also a Gaussian process, defined normally by $f(x) \sim GP(0,k(x,x^{\prime} ))$, where $k(x,x^{\prime} )$ is the kernel (covariance) function. In GPR, for known prior distributions of *f* and *y*, the posterior distribution of *f*_*new*_ can be computed. In our study, the kernel function *k*(*x*_*i*_, *x*_*i*_) is a radial basis functio which is

1.4 $$k({x}_{i},{x}_{j})={\sigma }_{f}^{2}\exp (-\frac{{({x}_{i}-{x}_{j})}^{T}({x}_{i}-{x}_{j})}{2{\sigma }_{l}^{2}}).$$

The predicted mean ${\bar{f}}_{new}$ is given by

1.5 $${\overline{f}}_{new}=K({x}_{new},X){[K(X,X)+{\sigma }^{2}I]}^{-1}y.$$

where *σ* ^*2*^ is the noise variance.

### Support vector regression (SVR)

SVR is an effective method of machine learning for regression problems^75^. The aim of SVR is to find a function to map a non-linear input space into a high dimensional feature space, so linear regression can be applied in this space. In SVR, the *ε*-insensitive loss function is commonly used, and a penalty factor *C* is chosen to control the balance between the model complexity and training errors. Like GPR, a kernel function is used to transform the data. The SVR optimization problem can be solved by quadratic programing algorithms, and the function to predict new data can be made by

1.6 $$f(x)=\sum _{i=1}^{N}({\alpha }_{i}-{\alpha }_{i}^{\ast })K(x,{x}_{i})+b.$$

where *α*, α* are Lagrange multipliers, and *K* (kernel function) is a radial basis function, which in this study is

1.7 $$K({x}_{i},{x}_{j})=\exp (-\frac{{({x}_{i}-{x}_{j})}^{T}({x}_{i}-{x}_{j})}{2{\gamma }^{2}}).$$

We next introduce the model fitting process. For the GLR model, we use all the data to fit the model. The *β* coefficients are calculated by the least squares approach. For the LASSO-GLR, the GPR and the SVR models, model fitting is performed by finding optimal parameters *λ* (LASSO-GLR), *σ*_*f*_ and *σ*_*l*_ (GPR), *C*, *ε*, and *γ* (SVR). In this study, 5- and 10-fold cross-validations are performed to find these parameters. Since the results of the two cross-validations show very slight difference (<1%), only the results of the 10-fold cross-validation are shown in this study. The overall data are randomly partitioned into ten folds. Model training is done with nine folds and validation is done with the remaining one. This process is repeated ten times with each fold used exactly once as the validation data. Furthermore, to investigate the effect of random partition on the predictive accuracy, we try four different random partitions to split data by assigning different seeds to random generator. The calculated predictive accuracies of the four partitions show slight differences (<1%). Therefore, we consider the model’s predictive accuracy difference caused by the random data choice is very small. In this study, the average predictive accuracies of the four random partitions are used.

In our study, the model fitting is performed with MATLAB statistical software^76^ under different initial settings based on the machine learning algorithm, training data and descriptor set.

## Results and Discussion

We have trained four models to predict TBR, which correspond to the four machine learning algorithms described above. We first predict the TBR of all the interfaces using the AMM and DMM. The details of the AMM and DMM predictions are described in ref. 5. For the AMM and DMM, the TBR can be written as

21 $$\frac{1}{R}=\frac{1}{2}\sum _{j}{c}_{i,j}{\int }_{0}^{\pi /2}{\alpha }_{i,j}\sin \theta \cos \theta d\theta {\int }_{0}^{{\omega }_{i}^{Debye}}\hslash \omega \frac{d{N}_{i,j}(\omega ,T)}{dT}d\omega .$$

where *R* is the TBR, *c*_*i*, *j*_ is the phonon propagation velocity in side *i* for phonons with mode *j* (longitudinal or transverse); *α*_*i*, *j*_ is the transmission probability of phonons from side *i* with mode *j*; *θ* is the angle between the wave vector of the incident phonon and the normal to the interface; and *ω* is the phonon frequency. ${N}_{i,j}(\omega ,T)\,\,$is the density of phonons with energy on side *i* with mode *j* at temperature *T*. The Debye cutoff frequency is given by

22 $${({\omega }_{i}^{Debye})}^{3}=\frac{1}{\frac{1}{3}{\sum }_{j}\frac{1}{{({c}_{i,j})}^{3}}}6{\pi }^{2}{(\frac{{N}_{atom}}{V})}_{i}.$$

where *N*_atom_/*V* is the atom number density of the crystal. For frequencies below $\,{\omega }_{i}^{Debye}$

2.3 $${N}_{i,j}(\omega ,T)=\frac{{\omega }^{2}}{2{\pi }^{2}{c}_{i,j}^{3}[exp(\hslash \omega /{k}_{B}T)-1]}.$$

where *k*_B_ is the Boltzmann constant.

In the AMM, *i* = 1 in Eqs (2.1), (2.2), and (2.3), and the transmission probabilities of phonons are calculated from the acoustic impedances.

2.4 $${\alpha }_{1\to 2}=\frac{4{Z}_{2}{Z}_{1}}{{({Z}_{1}+{Z}_{2})}^{2}}\,$$

where ${Z}_{i}={\rho }_{i}{c}_{i}$ is the acoustic impedance on each side of the interface, which equals to the product of the mass density and the phonon velocity.

In the DMM, the transmission probability of phonons are calculated by

2.5 $${\alpha }_{i,j}(\omega )=\frac{{\sum }_{j}{c}_{3-i,j}^{-2}}{{\sum }_{i,j}{c}_{i,j}^{-2}}.$$

**Figure 1:**
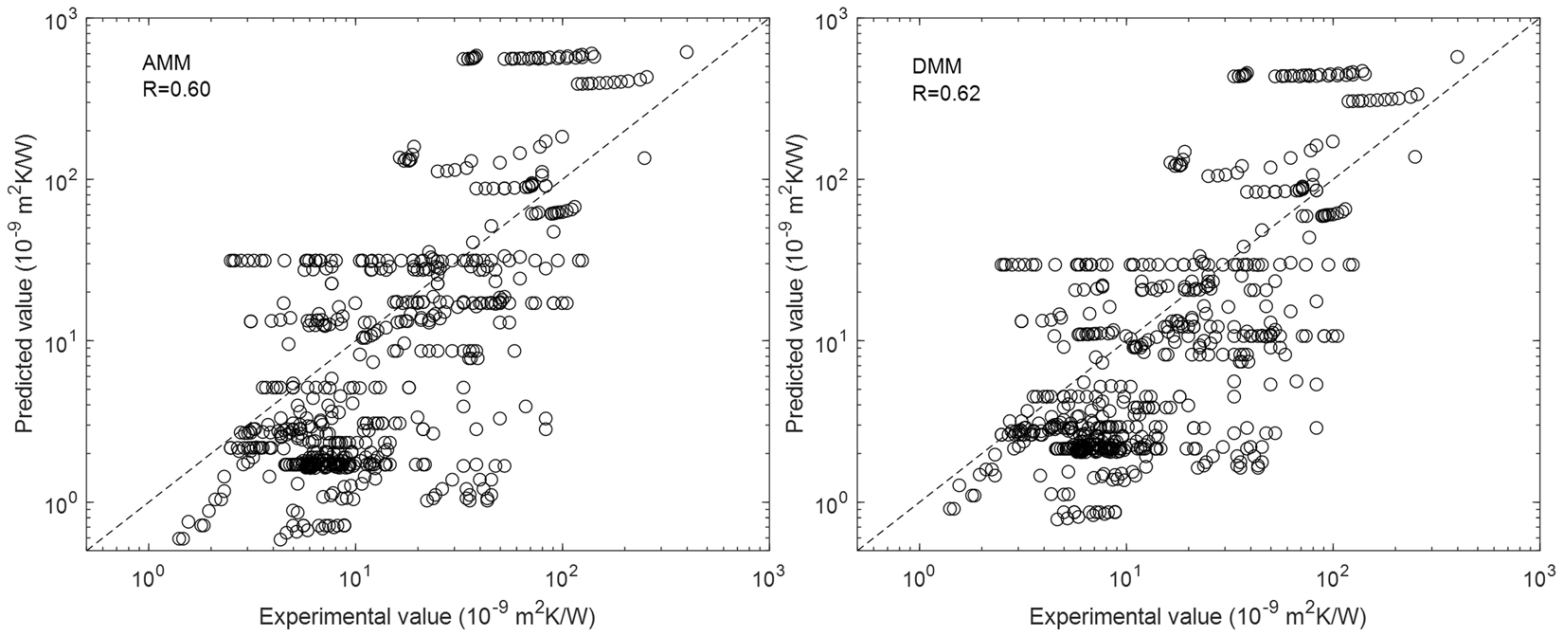
Correlation between the experimental values and the values predicted by the AMM and DMM.

The descriptors used for the AMM and DMM predictions are temperature, density, speed of sound (longitudinal and transverse), and unit cell volume, which we define as “AMM and DMM descriptors”. Figure 1 shows the correlation between the experimental values and the values predicted by the AMM and DMM. Two parameters, the correlation coefficient (*R*) and root-mean-square error (*RMSE*), are used to evaluate the predictive accuracy of the models. For the AMM, *R* and *RMSE* are 0.60 and 121.3, respectively. For the DMM, *R* and *RMSE* are 0.62 and 91.4, respectively. Thus both the AMM and DMM have low predictive accuracy, although the DMM is slightly better.

**Figure 2:**
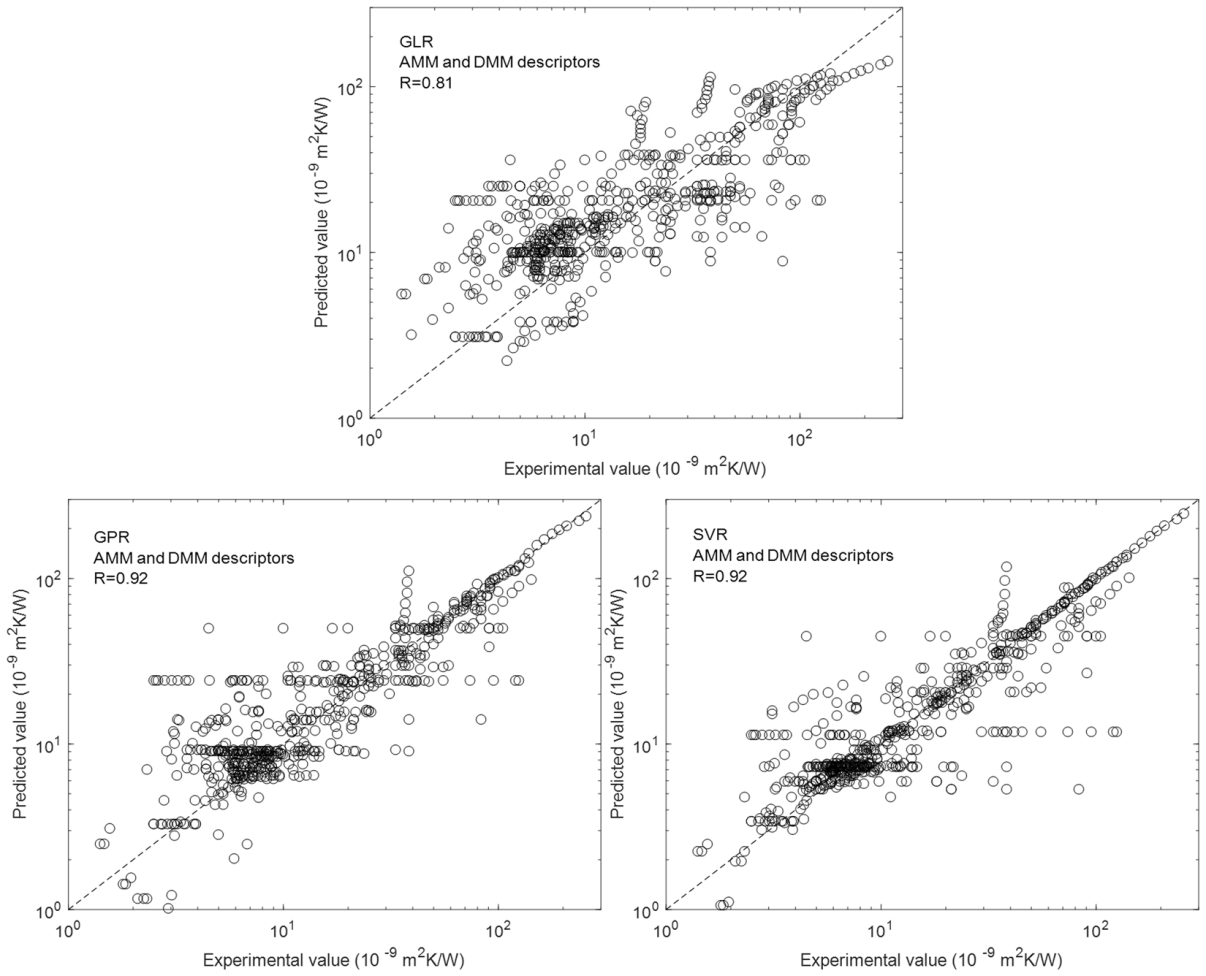
Correlation between the experimental values and the values predicted by the GLR, GPR, and SVR models using the AMM and DMM descriptors.

We next predict the TBR of all the interfaces using the machine learning models. It is known that the predictive accuracy of machine learning algorithms is strongly dependent on the selected descriptor sets^77^. In order to compare their predictions with those of the AMM and DMM, we have initially used the AMM and DMM descriptors. Figure 2 shows the correlation between the experimental and predicted values for the GLR, GPR, and SVR models. We next study the effects of descriptor set choice on the predictive accuracy of the machine learning models. First, we predict the TBR using all collected descriptors, because all collected measurement conditions and material properties may affect TBR. The values of *R* and *RMSE* for the GLR model and the average values of *R* and *RMSE* for the GPR and the SVM models are shown in Table 1. The results for the LASSO-GLR model are not given in Fig. 2 and Table 1, because the descriptors are different. As a result of model fitting, the LASSO-GLR model automatically selected 12 descriptors from the given 24, which are film thickness, film heat capacity, film melting point, film speed of sound (longitudinal), film thermal expansion, film unit cell volume, substrate thermal conductivity, substrate density, substrate speed of sound (longitudinal), substrate elastic modulus, substrate thermal expansion coefficient and substrate unit cell volume. We do not use this descriptor set in the following work because some important descriptors such as measurement temperature are missing, which is not reasonable from a physical point of view. The *R* and *RMSE* for the LASSO-GLR model are 0.89 and 15.8, respectively. ﻿For the GPR model, the average optimal σ_f_ and σ_l_ of the four random partitions are 176.22 and 5.56. For the SVR model, the average optimal C, ε, and γ are 987.15, 0.21 and 3.23. ﻿It is clear that all the machine learning models have much higher *R* and much lower *RMSE* than the AMM and DMM, indicating that all the machine learning models have a better predictive accuracy than the AMM and DMM. Among the machine learning models, the GPR and the SVR models have better performance than the GLR and the LASSO-GLR models.

**Table 1:** Comparison of *R* and *RMSE* predicted by different models using different sets of descriptors.

	AMM and DMM descriptors		All collected descriptors		“Reliable” descriptors	
	*R*	*RMSE*	*R*	*RMSE*	*R*	*RMSE*
AMM	0.60	121.3	N/A		N/A	
DMM	0.62	91.4	N/A		N/A	
GLR	0.81	20.3	0.9	15.4	0.81	20.1
GPR	0.92	13.2	0.96	9.6	0.96	9.4
SVR	0.92	13.9	0.95	10.4	0.96	9.9

However, model fitting using all collected descriptors is difficult because it is difficult to collect some of the descriptors, such as thermal conductivity, Debye temperature, speed of sound (longitudinal and transverse), elastic modulus, and bulk modulus, for all the materials from references. Also, different references usually give very different values for these material properties. For example, the measured thermal conductivity of crystalline AlN ceramics ranges from 17 Wm^−1^K^−1^ to 285 Wm^−1^K^−1^ ^78^. The measured thermal conductivity of nanoscale AlN thin films could be as low as 1.4 Wm^−1^K^−1^ ^79^. Such large difference may significantly affect the predictive accuracy. Therefore, we predict TBR using only “reliable” descriptors: measurement temperature, film thickness, heat capacity, melting point, density, and unit cell volume. These material properties are called “reliable” because they are easily collected from references and different references usually give similar values. With the “reliable” descriptors, we fit the GLR, GPR and the SVR models. The average optimal *σ*_*f*_ and *σ*_*l*_ are 106.68 and 2.59 for the GPR model. The average optimal *C*, *ε*, and *γ* are 916.51, 0.88 and 1.71 for the SVR model. Like the use of all collected descriptors, it can also be found that the GPR and the SVR models have better predictive accuracies than the GLR model.

**Figure 3:**
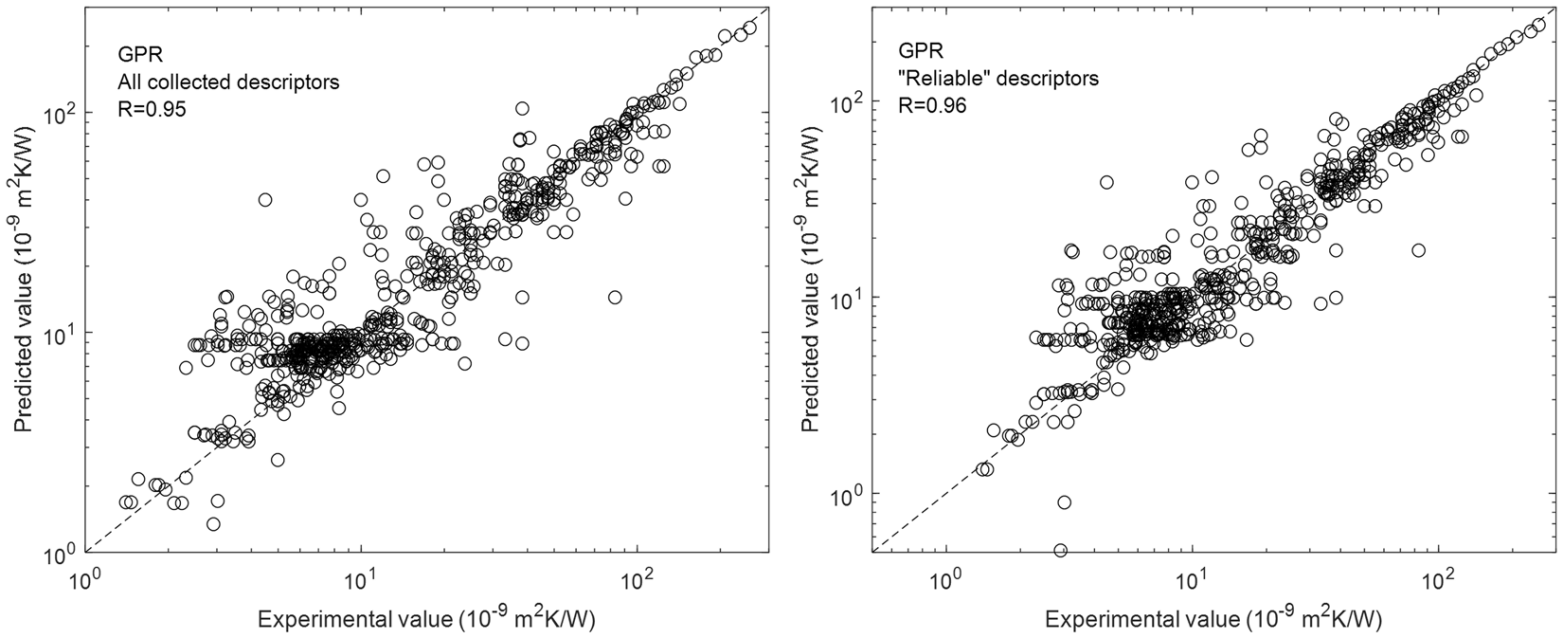
Correlation between the experimental values and the values predicted by the GPR model using the “reliable” descriptors and all collected descriptors.

Figure 3 shows the correlation between the experimental values and the values predicted by GPR model using the “reliable” descriptors and all collected descriptors. It is clear that the predictions using the “reliable” descriptors and all collected descriptors have better predictive accuracy than using the AMM and DMM descriptors. This indicates that other descriptors, in addition to those that are used in the AMM and DMM, also affect TBR. If we compare the AMM and DMM descriptors and the “reliable” descriptors, we find that measurement temperature, density, and unit cell volume are in both descriptor sets. However, film thickness, heat capacity, and melting point are included in the “reliable” descriptors instead of speed of sound. This indicates film thickness, heat capacity, and melting point descriptors improved the predictive accuracy. In order to know which one plays the most important role, we added film thickness, heat capacity, or melting point to the AMM and DMM descriptor set and predicted TBR using the GPR model. The predictions had *R* values of 0.96, 0.92, and 0.92, respectively. This indicates that of these three descriptors, film thickness plays the most important role. To further test our suggestion, we predicted TBR using the GPR model and replacing film thickness in the “reliable” descriptor set with thermal conductivity, Debye temperature, elastic modulus, bulk modulus, or thermal expansion. All the predictions without film thickness as descriptor show an *R* value of ~0.92, which is similar to that for the AMM and DMM descriptors and lower than the value of *R* when film thickness is included. These results indicate that film thickness is an important descriptor in the prediction of TBR. The dependence of TBR on film thickness has been found in our previous study of MD simulations on Si/Ge interface^80^. In that work, we used two different cell sizes, corresponding to film thickness of 50 and 100 nm, respectively. As a result, the 100 nm film has a lower TBR than the 50 nm one. The thickness dependence of TBR has also been observed in our experimental study on Au/sapphire interface^26^, where the TBR increases obviously when the grain size of Au decreases. The effect of film or grain size on TBR can be explained as that the phonon modes in the films are thickness or grain size dependent. Therefore, change in film thickness will change the phonon transmission probability at the interface as well as the TBR.

**Figure 4:**
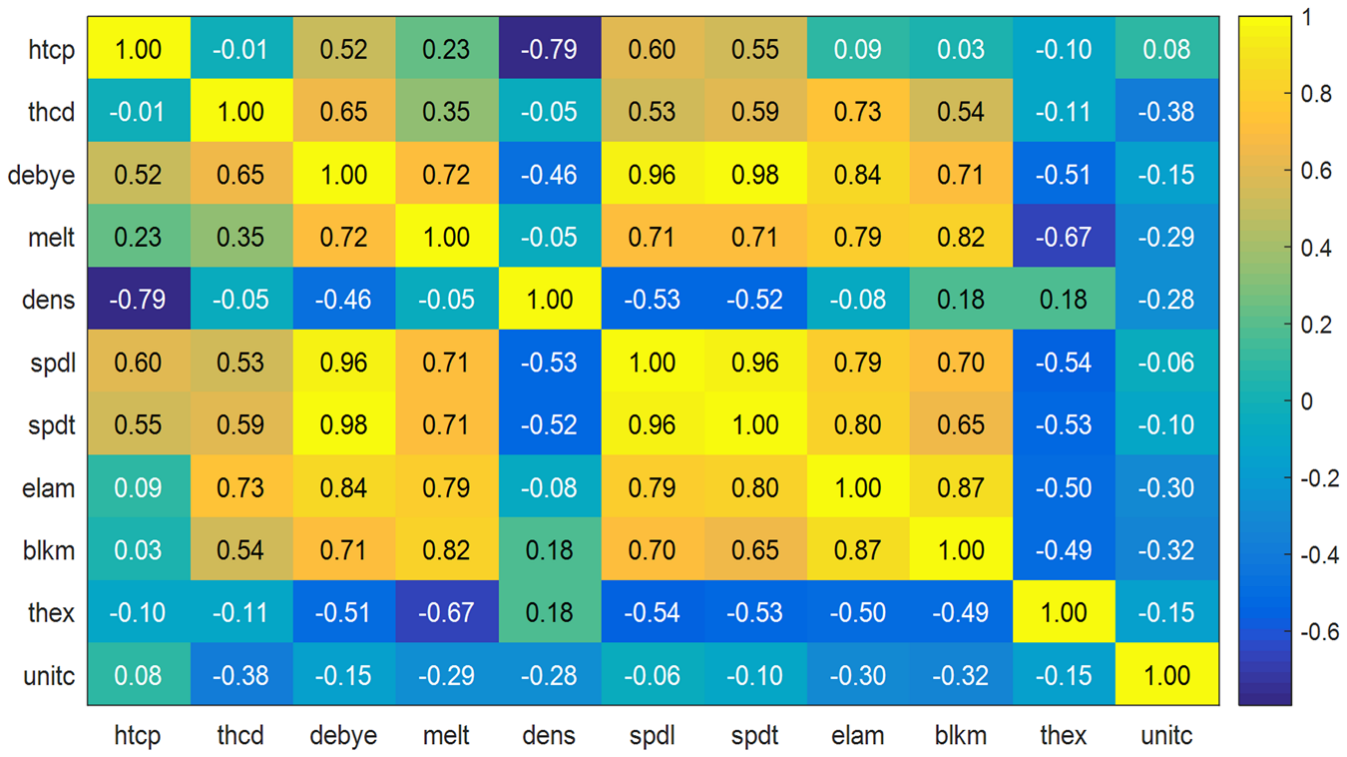
Pearson correlation coefficient map between different materials properties. htcp (heat capacity), thcd (thermal conductivity), debye (Debye temperature), melt (melting point), dens (density), spdl (speed of sound longitudinal), spdt (speed of sound transverse), elam (elastic modulus), blkm (bulk modulus), thex (thermal expansion coefficient), and unitc (unit cell volume).

Furthermore, the predictions using the “reliable” descriptors and all collected descriptors have similar predictive accuracy. This indicates that the use of more material properties as descriptors does not necessarily improve predictive accuracy. It is known that many material properties, including heat capacity, thermal conductivity, Debye temperature, melting point, density, speed of sound, elastic modulus, bulk modulus, and thermal expansion coefficient, are physically-correlated. Using the collected materials property data, we drew a Pearson correlation coefficient map between different materials properties (see Fig. 4). For example, the correlation coefficients between Debye temperature and speed of sound are higher than 0.96, which is in good agreement with the physical-correlation of the two descriptors. We, therefore, attribute the similar predictive accuracy of the two descriptor sets to the physical-correlation of these descriptors; it is thus unnecessary to use all collected descriptors simultaneously in the prediction of TBR.

## Conclusions

In summary, we have demonstrated a machine learning method for predicting TBR. We found that machine learning models have better predictive accuracy than the commonly used AMM and DMM. Among the trained models, the GPR and the SVR models have better predictive accuracy. Also, by comparing the prediction results using different descriptor sets, we found that film thickness is an important descriptor in the prediction of TBR. These results indicate that machine learning is a simple, accurate, and cost-effective method for the prediction of TBR. It can, therefore, be used to guide the discovery of interfaces with very low or very high TBR for the thermal management of high power micro- and opto-electronic devices and for the development of high efficiency thermal barrier coatings and thermoelectric materials.

## Electronic supplementary material

Supplementary Information
